# Comparison of arthroscopy‐ and fluoroscopy‐assisted minimally invasive approaches for acetabular fracture repair in dogs: An ex vivo study

**DOI:** 10.1111/vsu.70015

**Published:** 2025-09-08

**Authors:** Nikolaus Hubertus Huels, Johannes Siedenburg

**Affiliations:** ^1^ Clinic for Small Animals University of Veterinary Medicine Hannover Hannover Germany

## Abstract

**Objective:**

To describe and compare arthroscopy‐assisted (AA) with fluoroscopy‐assisted (FA) minimally invasive plate osteosynthesis (MIPO) for simple transverse acetabular fractures.

**Study design:**

Ex vivo cadaveric study.

**Sample population:**

A total of 10 canine cadavers (>20 kg) without coxofemoral joint disease.

**Methods:**

Pelvic computed tomography (CT) images were mirrored and three‐dimensional (3D) printed to create models for precontouring 2.7‐mm locking compression plates (LCP). Acetabula were randomly assigned to AA or FA MIPO groups and pelvis were prepared for stabilization by standardized osteotomies of the pubic, ischial and acetabular bones. In the AA group, fracture reduction was arthroscopically confirmed, and precontoured plates were applied via small approaches to the ilium and ischium. In the FA group, reduction was guided fluoroscopically. Surgical time, incision length, procedural complications, and feasibility were recorded. Postprocedural CT scans measured fracture gap, step formation, medio‐lateral displacement and pelvic angulation. Necropsy assessed iatrogenic injuries.

**Results:**

MIPO was successful for all 20 acetabula. Mean procedure time and incision length were not significantly different between groups. Mean fracture gaps and step defects were <1 mm in both groups. Medio‐lateral displacement exceeded 1 mm in the FA group (median 1.08 mm) compared to 0.74 mm in the AA group. Low coronal angles (<5°) were consistent across procedures. Sciatic nerve injury occurred in one case per group. Minor superficial cartilage damage was common.

**Conclusion:**

Arthroscopy‐assisted MIPO was feasible for simple acetabular fractures, resulting in anatomic (6/10) or near‐anatomic (4/10) reductions.

**Clinical significance:**

Further studies and clinical experience are necessary before recommending AA as an alternative for open approaches.

## 1 INTRODUCTION

Acetabular fractures in dogs are common injuries resulting from high‐impact trauma such as vehicular accidents and often require surgical intervention. Direct surgical repair is used to address these fractures in cases where anatomic reconstruction is possible. Open approaches often involve osteotomies of the greater trochanter or tenotomies of the gluteal muscles to access the acetabulum. These approaches can cause significant soft tissue trauma and carry the risk of iatrogenic sciatic nerve injury.^1^, ^2^, ^3^ Because of the rigidity and complexity of the articular surface, anatomic reconstruction can be challenging but is considered to be crucial for achieving favorable clinical outcomes and restoring limb function.^4^, ^5^, ^6^, ^7^, ^8^

In recent years, minimally invasive osteosynthesis (MIO), such as minimally invasive plate osteosynthesis (MIPO), has gained popularity in both human and veterinary medicine. Reported advantages of MIO over conventional open approaches have been identified in earlier return to function with reduced morbidity, likely due to reduced tissue trauma and preservation of blood supply to the fracture fragments.^6^, ^9^, ^10^, ^11^, ^12^ Accordingly, MIPO has shown positive clinical outcomes and facilitates fast recovery, particularly for simple fractures of long tubular bones.^13^, ^14^, ^15^ Using a locking plate in the function of a bridging plate applied under fluoroscopic assistance has been described for the repair of canine acetabular fractures.^16^ The treatment of complex fractures, such as comminuted fractures, highly dislocated fractures and fractures involving the articular surface that require precise anatomical reduction, is challenging. To address these issues, various techniques have been developed to enhance the success of minimally invasive surgery.

The integration of three‐dimensional (3D) technology has played a significant role in advancing minimally invasive fracture treatment.^17^, ^18^ By utilizing computed tomography (CT) scans to generate STL files, fractures can be accurately reconstructed, or the contralateral bone can be mirrored to 3D print bone models.^16^ This advancement allows for preoperative contouring of implants, reduces surgical and anesthesia time, and aids in fracture reduction.^19^

Although minimally invasive approaches for articular fractures typically employ fluoroscopic visualization, intraoperative arthroscopy has emerged as an alternative technique.^6^ Although intraoperative arthroscopy is regularly utilized in human medicine for joint fractures and is well described for acetabular fractures, the application of arthroscopic assistance for acetabular fractures in veterinary medicine has only been reported anecdotally.^6^, ^20^, ^21^ Indications for hip joint arthroscopy include minimally invasive placement of a toggle rod for treating coxofemoral luxation, as well as its use as a diagnostic tool prior to performing pelvic osteotomies in juvenile dogs with hip dysplasia.^22^, ^23^

To the best of our knowledge, MIPO for central acetabular fractures with 3D printing has been described for fluoroscopy; however, there are no reports using arthroscopic assistance for fracture reduction.^16^ The aim of this cadaveric study was to evaluate the feasibility and safety of using AA for MIPO in simple transverse central acetabular fractures and to achieve anatomic fracture repair with small fracture gaps (<1 mm), small step defects (<1 mm), small medio‐lateral displacements (<1 mm) and minimal changes in sagittal and coronal angles (<5°).^24^ Furthermore, we hypothesized that arthroscopy‐assisted MIPO results in better postoperative reduction than MIPO under fluoroscopic guidance. Second, we hypothesized that arthroscopy would result in iatrogenic cartilage damage and increased surgical time compared to fluoroscopy. An ex vivo cadaveric study was conducted to evaluate the feasibility, safety, and accuracy of two minimally invasive fracture repair methods: fluoroscopy and arthroscopy‐assisted techniques.

## 2 MATERIALS AND METHODS

An ex vivo cadaveric study was conducted to evaluate the feasibility, safety, and accuracy of two minimally invasive fracture repair methods: fluoroscopy‐ and arthroscopy‐assisted techniques. An unstable mid‐acetabular fracture model was created by performing pubic, ischial, and simple transverse central acetabular osteotomies under fluoroscopic guidance.^16^ The experimental protocol was approved by the Research Ethics Committee (University of Veterinary Medicine Hannover; ethics vote: TiHo_EA_13_20–24).

### 2.1 Specimen

A total of 10 canine cadavers weighing >20 kg were used in this study. From each cadaver, one acetabulum was randomly allocated to either the arthroscopy (AA)‐ or fluoroscopy (FA)‐assisted MIPO group. The dogs were euthanized for reasons unrelated to the study, and the owners confirmed their use for scientific purposes. Dogs with anamnestic signs of hip dysplasia or those that had previously undergone hip joint surgeries were excluded.

### 2.2 Preprocedure imaging

CT imaging of each pelvis was conducted within 24 h postmortem, and the cadavers were subsequently frozen at −18°C and thawed at room temperature 48 h prior to the surgical procedure. The scans were acquired using a spectral detector CT (Philips IQon Spectral CT, Philips Healthcare, Hamburg, Germany) with a 0.5 mm slice thickness with a kilovolt peak (kVp) of 120 and an x‐ray tube current of 205 mA. Additionally, the preoperative CT scans were evaluated for signs of coxofemoral disease.

### 2.3 3D modeling and printing

3D modeling and creation of the STL files for printing was performed as previously described by Dalton et al.^16^ Briefly, the CT images of each pelvis were segmented and 3D reconstructed using digital imaging and communications in medicine (DICOM) files, which were imported into the Materialize Medical Imaging Software Suite (Mimics 22.0 and 3‐matic 17.0; Materialize NV, Leuven, Belgium). The 3D visualizations, referred to as masks, were created using the Hounsfield unit density range for bone (+258 to +2455). To isolate the pelvises from the adjacent bony structures, the masks were manually refined by identifying the regions of interest. Any imperfections were corrected using wrapping and hole filling algorithms. The 3D models were constructed using Mimics software (Materialize Mimics 22.0 Medical, Materialize NV) and then imported into 3‐matic software (Materialize 3‐matic 17.0 Medical, Materialize NV). The pelvises were then mirrored using an algorithm. The digital pelvic model was subsequently divided into hemipelvises along the pelvic symphysis using a trimming algorithm. The STL files of the hemipelvises were sent to an external company (Navicos GmbH, Hannover, Germany) for 3D printing at their original anatomical size. Printing was performed using a pulver‐based selective laser sintering (SLS) printer. To ensure model accuracy, the printed specimens were evaluated using a strip laser scanner, and the process complied with DIN EN ISO 13485, a quality standard for medical devices in human medicine. This ensured high anatomical fidelity of the 3D models, allowing for reliable preoperative planning and simulation.

**FIGURE 1:**
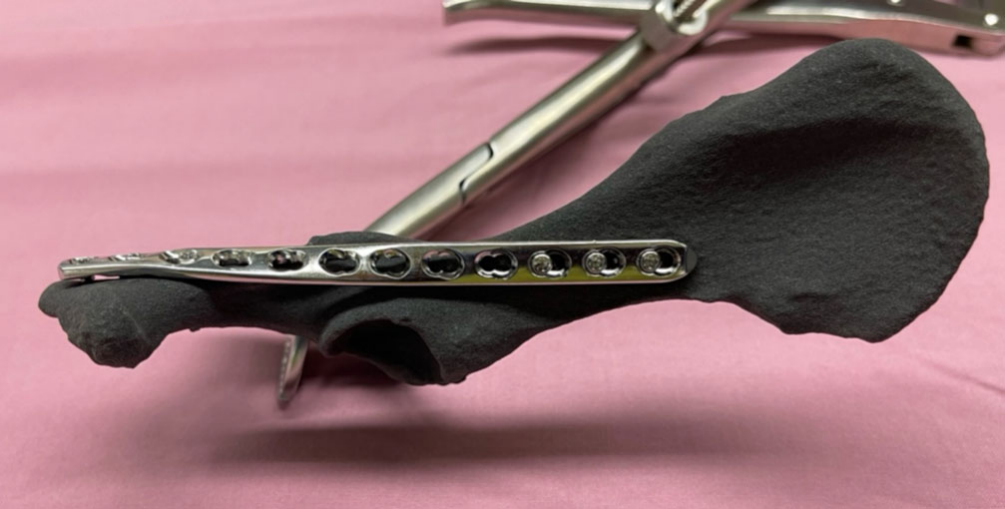
Left mirrored hemipelvis of cadaver 5 with a precontoured 2.7 mm locking compression plate fixated with three locking screws cranial and three locking screws caudal.

For each hemipelvis, a 2.7 mm LCP (Synthes, Oberdorf, Switzerland) was pre‐contoured based on the mirrored 3D printed model. The length of the plate was chosen depending on the size of the pelvis to allow for the placement of a minimum of three bicortical locking screws per fragment (Figure 1).

### 2.4 Surgical technique

#### 2.4.1 Arthroscopy‐assisted

Arthroscopy‐assisted minimally invasive fracture repair was performed using either a standard 2.4 mm 30° scope (Synergy UDH4 4 K Imaging System, Arthrex, Naples, Florida) or a 1.9 mm needle arthroscope (Nanoscope, Arthrex) through a 2.2 mm fluid port. The camera, with a resolution of 400 × 400 pixels, was linked to an imaging console featuring a 13‐in. display. The choice between the needle arthroscope and the conventional 2.4 mm arthroscope was based on availability.

Each cadaver was positioned in lateral recumbency, and the area over the hip joint was clipped and draped. The hindlimbs were brought into an ideal position for coxofemoral arthroscopy, with slight limb adduction, 30° of hip flexion, and the stifles in a neutral position. The arthroscopic portal was placed approximately 5 mm cranial and 15 mm proximal to the greater trochanter.^16^ The joint space was expanded by instilling saline using a 20‐gauge needle inserted perpendicular to the limb dorsal to the greater trochanter. A portal was created at the 12 o'clock position by making a small incision with a #11 scalpel blade and, if needed, with a mosquito clamp. Egress portals were established with a 20‐gauge needle, if needed, placed at the 5 o'clock position for the right hip and the 7 o'clock position for the left hip. Fluid flow was maintained with a pressurized fluid pump (DualWave, Arthrex) set to 60 mmHg in the standard arthroscopy group and with passive pressure using infusion bags with a pressure manometer set to 60 mmHg in the needle arthroscopy performed procedures.

After the port was established, full exploration of the joint was performed. The visualization of the intra‐articular structures was assessed similarly to a previous report by Kim et al.^17^ A score of “excellent” was given if all structures were visible, “good” if 1–3 areas were not visible, “poor” if 4–6 areas were not visible, and “fail” if >6 areas were not visible. The elevated structures were slightly modified as follows: acetabular rim and notch, femoral head, fracture line, ligament of the femoral head, articular capsule, and transverse acetabular ligament. Complications such as the loss of portals or impingement of the scope during fracture reduction were documented.

#### 2.4.2 Fluoroscopy

Fluoroscopy was performed using a C‐arm (Philips BV Libra C‐Bogen; Philips Healthcare). The cadaver was placed on a table suitable for fluoroscopic procedures that allowed for manipulation of the dog. The number of images and duration of permanent fluoroscopy used during the procedure were documented.

#### 2.4.3 Randomization

For each cadaver, one randomly selected acetabulum (right or left) was stabilized using a randomly assigned surgical technique (AA or FA), determined using the RAND function in Microsoft Excel. After the first procedure, the contralateral hemipelvis was prepared and the acetabulum stabilized using the other technique.

#### 2.4.4 Surgical approach

Small approaches were performed cranial to the ilium and caudal to the tabula ossis ischii. To approach the ilium the subcutaneous tissue, gluteal fat, and superficial fascia were incised and retracted along the margins of the skin incision. The deep gluteal fascia was incised over the ventral margin of the ilium to allow separation of the septum between the middle gluteal muscle and the tensor fascia lata muscle. The gluteal muscles were elevated and retracted cranially.^1^ To assess the tabula ossis ischii the internal obturator muscle was elevated from the tabula, and a small incision was made in the semitendinosus muscle to grasp the ischial tuberosity with bone forceps. Cranial and caudal approaches were connected by blunt dissection, creating a periosteal tunnel. The LCP was inserted from the caudal ischial approach in a cranial direction, and care was taken to place the plate close to the bone to maintain its position beneath the sciatic nerve.^24^ Once the approaches were connected, the plate was applied, and each fragment was grasped with bone forceps. The reduction was performed with arthroscopic or fluoroscopic assistance. The fracture was temporarily held in reduced position manually with the forceps. As soon as satisfactory fracture reduction was achieved, the plate was fixed with three bicortical locking screws per fragment, alternating between caudal and cranial fixations. All procedures were performed by the same ECVS diplomate (JS) and an ECVS resident (NH).

### 2.5 Measurement outcomes

Procedural parameters included incision lengths of the cranial and caudal approach, surgical time of the approaches, time to creation of the arthroscopic portals and the total procedure duration. Intraoperative complications were also documented. Fracture gaps, step defects and changes in pelvic angulation (coronal and sagittal angles) were calculated as described by Dalton et al.^16^ Additionally we calculated the medio‐lateral displacement. Quality of final fracture reduction was assessed as anatomic, near‐anatomic, or nonanatomic^24^ (Table 1).

**TABLE 1:** Modified classification system based on Boswell et al. for assessment of fracture reduction quality, including medio‐lateral displacement and changes in pelvic angulation.^24^

Quality of reduction	Fracture gap, step defect and medio‐lateral displacement	Changes in pelvic angulation (sagittal and coronal angles)
Anatomic	<1 mm displacement	<5°
Near‐anatomic	1–2 mm displacement	<5°
Nonanatomic	≥2 mm displacement	>5°

#### 2.5.1 Post‐procedure imaging, assessment data analysis

**FIGURE 2:**
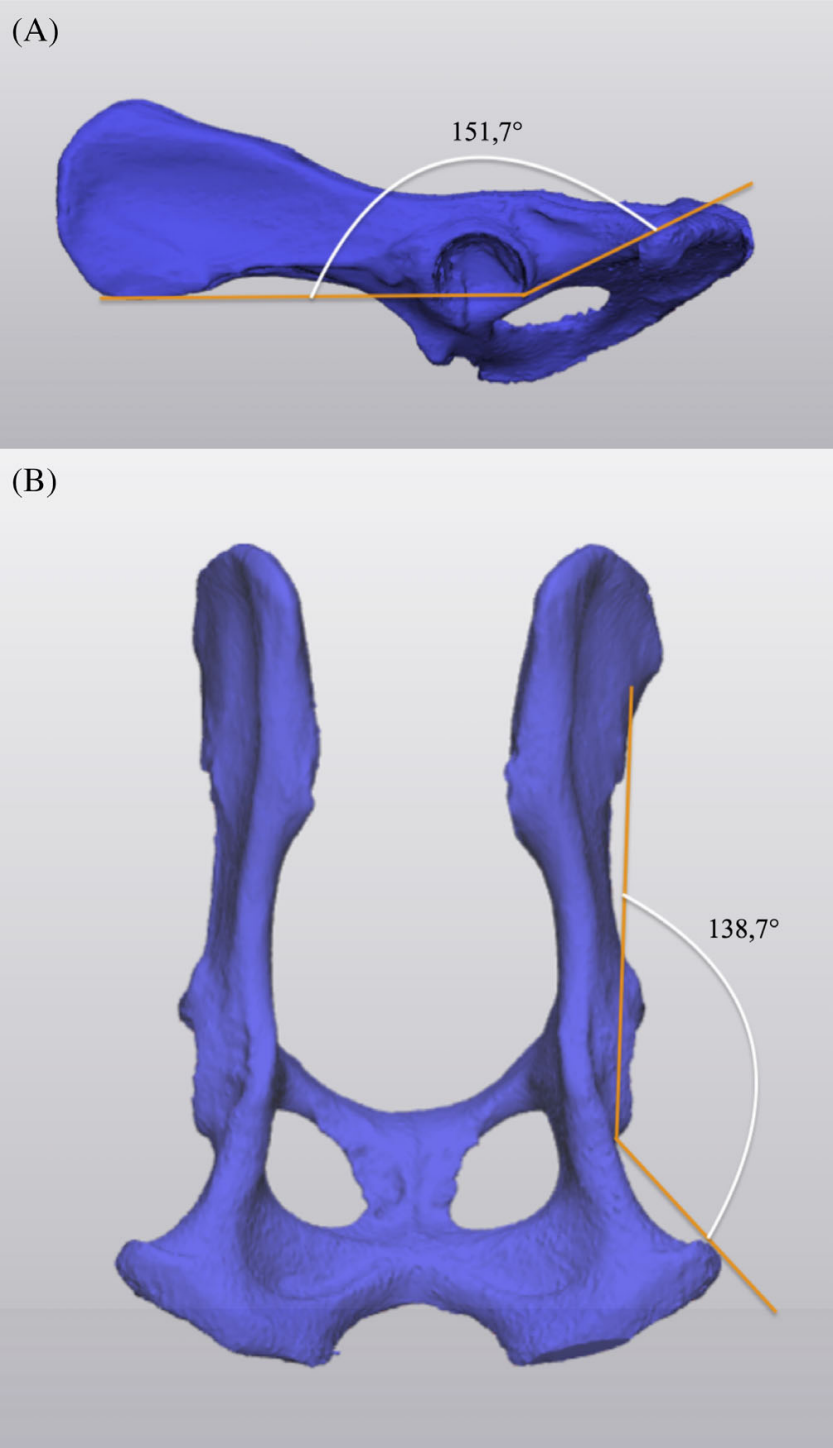
Exemplary measurement of preoperative sagittal (A) and coronal (B) angles using three‐dimensional (3D)‐rendered pelvic models, following the method described by Dalton et al.^16^ The angles were determined in their respective planes, utilizing reference lines from the ischiatic tuberosity and the cranial ventral iliac spine, which intersected at the caudal acetabular edge.

Following the surgical procedure, CT was performed with the same settings as for preoperative scans with the addition of metal artifact reduction (MAR) applied using the spectral CT to minimize interference from metallic implants and enhance image quality. Fracture gaps and step defects were measured as the largest horizontal and vertical separation plane of the bones in the sagittal using the distance tool in Horos (Horos Project, Geneva, Switzerland).^16^ Additionally, the medio‐lateral displacement of the fracture was measured in the coronal plane. Pelvic angulation (sagittal and coronal angles) was measured as previously described.^16^ (Figure 2) Finally, the specimens were anatomically dissected for the subjective evaluation of sciatic nerve damage and iatrogenic cartilage damage in the arthroscopy group. High‐resolution photographs (Sony Alpha 7 RIV, paired with Sony 70–200 GM II, Sony, Tokyo, Japan) were taken of each acetabulum and femoral head in the arthroscopy group to evaluate the presence of iatrogenic cartilage damage and to categorize them as superficial or full thickness, depending on whether the subchondral bone was exposed. The extent of cartilage damage was calculated by JS.

### 2.6 Statistical analysis

Data are reported using descriptive statistics. Statistical analyses were performed using Microsoft Excel (Microsoft Corporation, Redmond, Washington). Normality was assessed using the Shapiro–Wilk test, and Q‐Q plots. Normally distributed data are reported as mean (SD), not‐normally distributed data are reported as median (range). Data were compared using a parametric (*t*‐test) or a non‐parametric test (Mann–Whitney U test) depending on data normality.

## 3 RESULTS

### 3.1 Sample size

A total of 10 cadavers were used in this study. All specimens weighed >20 kg and were medium‐sized. In each cadaver, one acetabular fracture was repaired using AA‐MIPO, while the contralateral fracture was stabilized with FA‐MIPO, thereby enabling the comparison of both techniques within the same subject. No specimens were excluded from the study.

### 3.2 Surgery

**FIGURE 3:**
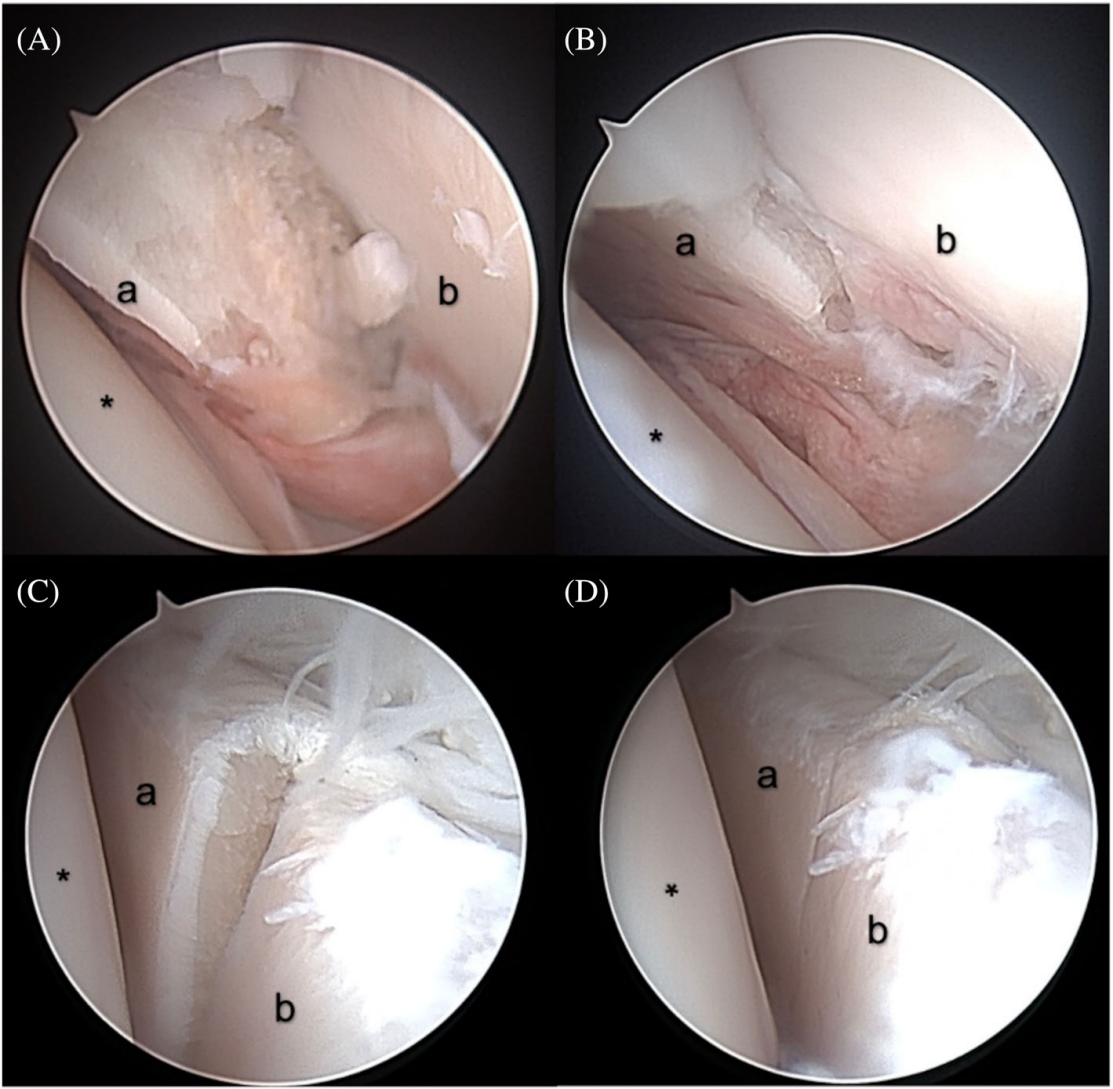
Arthroscopic images of the left hip of cadaver 7 using a 2.4 mm 30° scope (A: Prior to reduction, B and C during reduction, D: Final reduction of the fracture). The images are labeled as follows: “*” denotes the femoral head, “a” indicates the cranial part of the acetabulum, and “b” marks the caudal part of the acetabulum.

**FIGURE 4:**
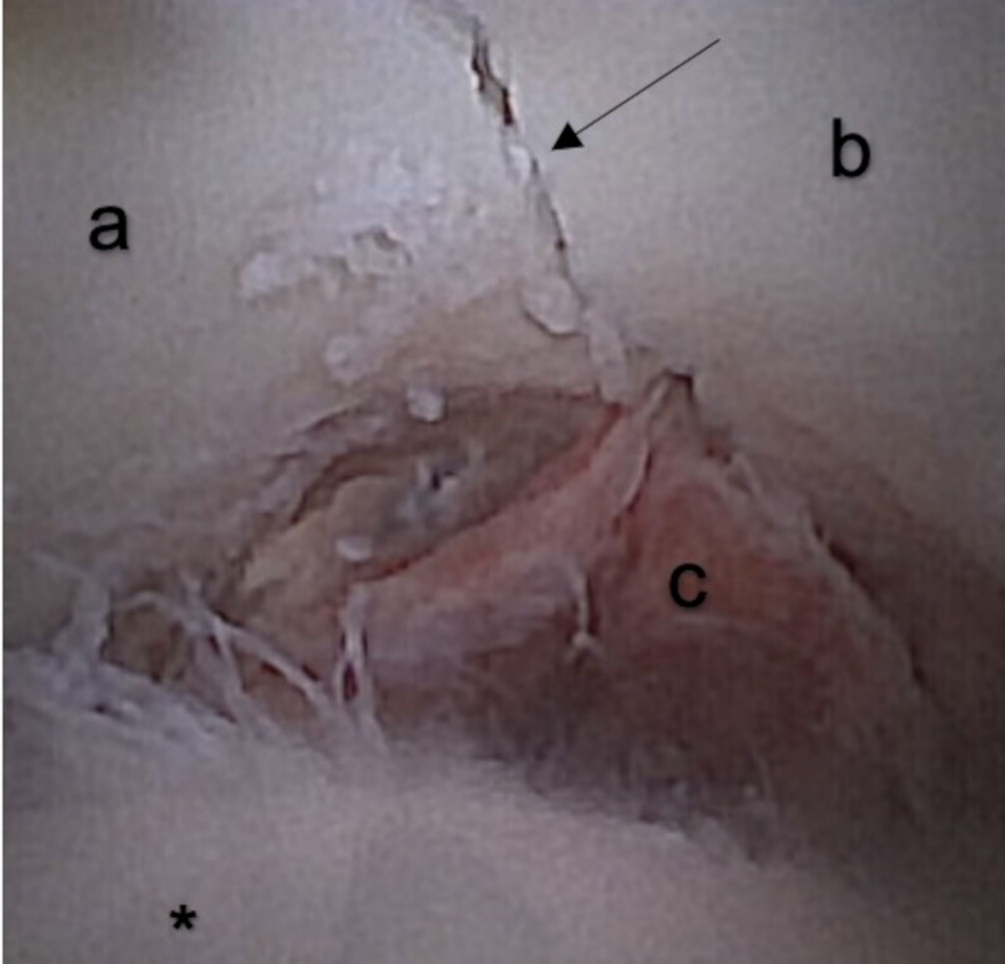
Arthroscopic image of the left hip of cadaver 1 post reduction. The images are labeled as follows: “*” denotes the femoral head, “a” indicates the cranial part of the acetabulum, “b” marks the caudal part of the acetabulum, “c” marks the ligamentum of the fermoral head and the arrow points at the fracture line.

Fracture stabilization by application of a 2.7 mm LCP with three bicortical screws per fracture segment was feasible using both techniques. Correct screw positioning was verified on post‐procedural CT scans. Arthroscopy was associated with intraoperative complications. In two cases (2/10), loss of the arthroscopic port was documented, and in another two cases, the scope impinged between the femoral head and acetabulum during manipulation of the fragments, making fracture reduction challenging. Three cases in the arthroscopy group were treated using a standard arthroscopy 2.4 mm 30° scope (Synergy UDH4 4 K Imaging System, Arthrex) (Figure 3), and seven cases were treated using needle arthroscopy (Nanoscope, Arthrex) (Figure 4).

### 3.3 Outcome measurements and incision length

**FIGURE 5:**
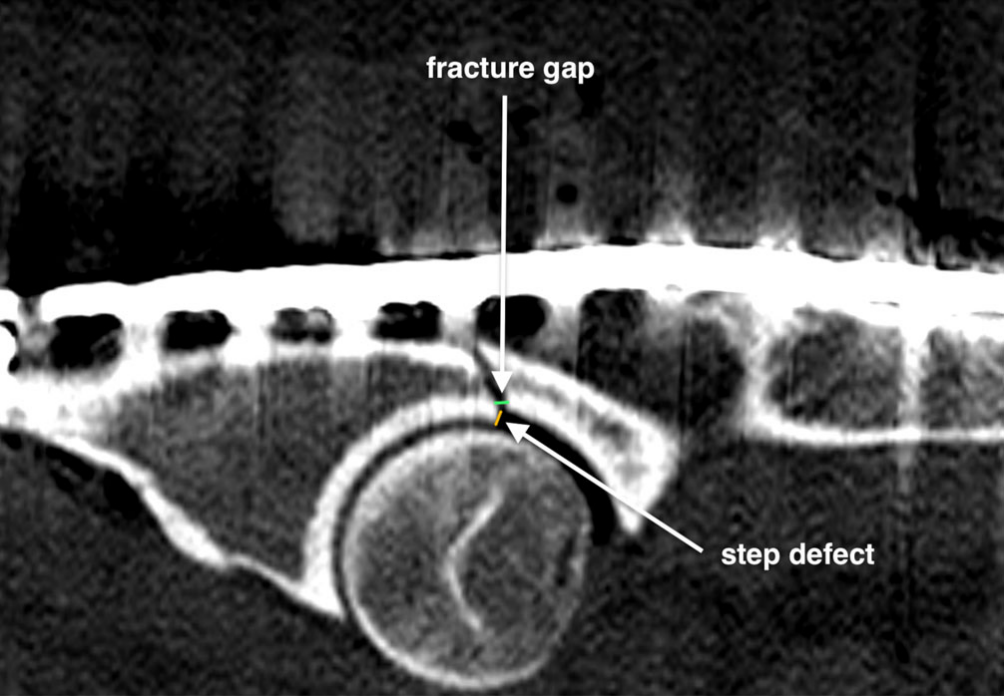
Sagittal view of the post‐procedure computed tomography scan in bone window. The fracture gap was evaluated as the widest point of separation between the fracture edges, whereas the step defect was determined by measuring the longest segment of misalignment along the articular surface.

**FIGURE 6:**
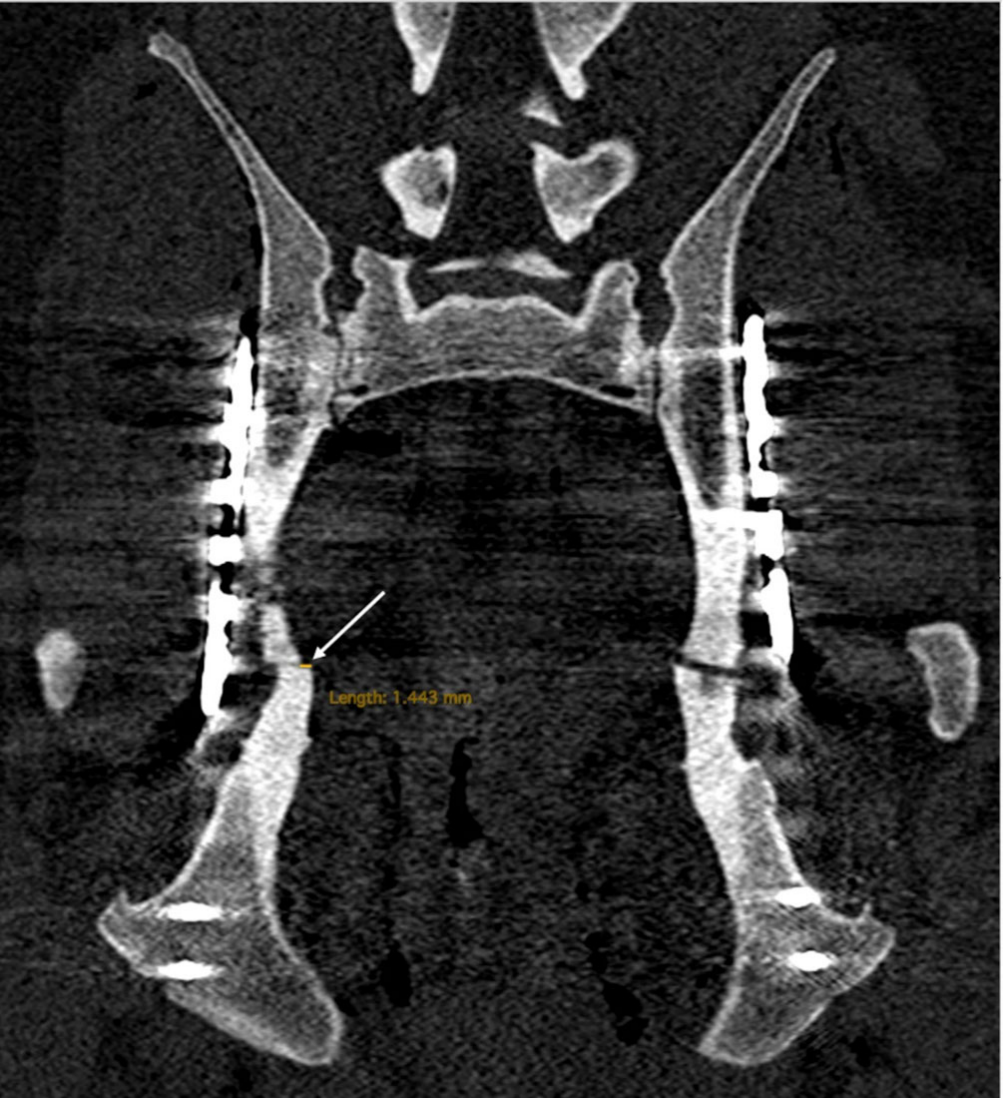
Coronal view of the post‐procedure computed tomography scan in bone window. The arrow indicates the location of the medio‐lateral displacement.

The mean fracture gap was 0.6 ± 0.3 mm in the AA group and 0.9 ± 0.4 mm in the FA group, both measuring less than 1 mm, with no significant difference between the groups (*p* = .1). The mean step defect was 0.4 ± 0.2 mm in the AA group and 0.7 ± 0.2 mm in the FA group, both measuring less than 1 mm, with no significant difference between the groups (*p* = .15) (Figure 5). The medio‐lateral displacement had a mean of 0.75 mm ± 0.62 and a median (IQR) of 0.74 mm (range: 0.28–1.0) in the AA group, whereas the FA group had a median (IQR) of 1.08 mm (range: 0.17–1.78) and a mean of 1.30 mm ± 1.468. Nevertheless, the difference between groups was not statistically significant (*p* = .4). (Figure 6). The mean change in the coronal angle was 3.17° (± 0.71°) in the AA group and 2.04° (±1°) in the FA group. The mean change in sagittal angles was 2.02° (±1.03°) with arthroscopy and 2.12° (±1.43°) with fluoroscopy (Tables 2, 3, 4). Comparing AA and FA procedures, statistically significant differences existed in the mean total surgical time (1:01:36 ± 17:23 min:s with AA and 39:49 ± 13:16 min:s with FA; *p* = .004) (Tables 5, 6), as well as total procedure time (excluding the time for the cranial and caudal approach, 45:37 ± 16:08 min:s with AA and 31:01 ± 12:39 min:s with FA; *p* = .005); however, no statistically significant differences were detected for reduction and fixation time (excluding the time for the arthroscopic port placement, 42:36 min:s with AA and 31:01 ± 12:39 min:s with FA; *p* = .07). Comparison of AA and FA regarding cranial (6.81 cm ± 1.81 cm for AA and 7.01 cm ± 1.95 cm for FA, *p* = .1) and caudal (4.48 cm ± 0.815 cm for AA and 4.35 cm ± 0.81 cm for FA, *p* = .07) incision lengths showed no significant differences. The quality of final fracture reduction was classified as anatomic in 6/10, near‐anatomic in 4/10, and nonanatomic in 0/10 acetabula treated with AA‐MIPO, compared to anatomic in 4/10, near‐anatomic in 5/10, and non‐anatomic in 1/10 acetabula treated with FA‐MIPO.

**TABLE 2:** Postoperative assessment – arthroscopy.

Cadaver	Scope used	Fracture gap (mm)	Step defect (mm)	Medio‐lateral displacement (mm)	Sagittal angle (°) preoperative	Sagittal angle (°) postoperative	Sagittal angle (°) change	Coronal angle (°) preoperative	Coronal angle (°) postoperative	Coronal angle (°) change	Sciatic nerve injury	Cartilage damage	Complication
1	Needle arthroscope, 0°	0.362	0.509	0.116	139.16	140.0	0.84	135.81	133.68	2.13		Femoral head <10%, acetabulum <10%	
2	Needle arthroscope, 0°	0.834	0.422	1.010	139.43	140.65	1.22	156.93	154.49	2.44		Femoral head <10%	
3	Needle arthroscope, 0°	0.410	0.218	1.499	144.96	141.51	3.45	141.35	138.85	2.5			Impingement through the scope
4	Needle arthroscope, 0°	0.542	0.623	1.872	143.27	140.44	2.83	144.34	141.45	2.89		Femoral head <10%, acetabulum <10%	
5	Needle arthroscope, 0°	0.219	0.725	0.155	146.17	145.19	0.98	142.84	146.08	3.24			Loss of arthroscopic ports
6	Needle arthroscope, 0°	0.752	0.112	0.101	145.01	146.24	1.23	133.16	137.28	4.12		Femoral head <10%	
7	2.4 mm, 30°	0.311	0.286	0.275	137.41	139.75	2.34	144.92	141.27	3.65	Sciatic nerve impingement, nerve under the plate		
8	2.4 mm, 30°	0.689	0.364	0.735	141.70	140.49	1.21	133.43	137.64	4.21			
9	2.4 mm, 30°	0.988	0.201	1.082	141.65	138.32	3.33	142.04	145.02	2.98		Femoral head <10%, acetabulum <10%	Impingement through the scope
10	Needle arthroscope, 0°	1.215	0.829	0.672	151.79	149.03	2.76	138.72	142.28	3.56		Femoral head <10%, acetabulum <10%	Loss of arthroscopic ports, difficulty to visualize cranial joint compartment
Mean		0.632	0.429	0.752	143.055	142.162	2.019	141.354	141.804	3.172			
±SD		0.321	0.239	0.615	4.183	3.443	1.025	6.948	5.800	0.7110			

**TABLE 3:** Postoperative assessment – fluoroscopy.

Cadaver	Fracture gap (mm)	Step defect (mm)	Medio‐lateral displacement (mm)	Sagittal angle (°) preoperative	Sagittal angle (°) postoperative	Sagittal angle (°) change	Coronal angle (°) preoperative	Coronal angle (°) postoperative	Coronal angle (°) change	Sciatic nerve injury
1	0.492	0.194	0.252	139.36	139.01	0.35	136.08	134.78	1.35	
2	1.575	0.132	4.901	139.87	140.21	0.34	156.81	155.92	0.89	
3	0.713	0.446	0.778	147.43	143.93	3.45	140.60	141.83	1.23	
4	0.696	0.290	1.448	142.98	140.42	2.56	142.55	146.31	3.76	
5	0.312	0.789	0.102	145.83	142.41	3.42	143.00	143.98	0.98	
6	1.298	1.807	0.145	144.52	146.86	2.34	129.45	131.47	2.02	
7	0.945	1.142	1.988	137.23	141.8	4.57	144.46	141.59	2.87	Sciatic nerve impingement, nerve under the plate
8	0.695	0.223	0.104	141.86	143.97	2.11	131.97	135.19	3.22	
9	1.447	1.865	1.389	136.40	135.51	0.89	136.49	139.05	2.56	
10	0.974	0.437	1.896	145.84	146.97	1.13	121.22	122.79	1.57	
Mean	0.9147	0.732	1.300	142.132	142.109	2.116	138.263	139.291	2.045	
±SD	0.415	0.239	1.468	3.825	3.540	1.434	9.686	9.007	1.006	

**TABLE 4:** Combined postoperative assessment.

Parameter	AA group (mean ± SD)	FA group (mean ± SD)
Fracture gap (mm)	0.632 ± 0.321	0.915 ± 0.415
Step defect (mm)	0.429 ± 0.239	0.732 ± 0.239
Medio‐lateral displacement (mm)	0.752 ± 0.615	1.300 ± 1.468
Sagittal angle preop (°)	143.06 ± 4.18	142.13 ± 3.83
Sagittal angle postop (°)	142.16 ± 3.44	142.11 ± 3.54
Sagittal angle change (°)	2.02 ± 1.03	2.12 ± 1.43
Coronal angle preop (°)	141.35 ± 6.95	138.26 ± 9.69
Coronal angle postop (°)	141.80 ± 5.80	139.29 ± 9.01
Coronal angle change (°)	3.17 ± 0.71	2.05 ± 1.01
Sciatic nerve injury (n)	1/10 (impingement)	1/10 impingement)
Quality of final fracture reduction	Anatomic (6/10), near‐anatomic (4/10), non‐anatomic (0/10)	Anatomic (4/10), near‐anatomic (5/10), non‐anatomic (1/10)

**TABLE 5:** Surgical measurements and duration of hip arthroscopy group.

Cadaver	Scope used	Caudal approach length (cm)	Caudal approach time (hh:mm:ss)	Craniolateral approach length (cm)	Craniolateral approach time (hh:mm:ss)	Placement of arthroscopic ports time (hh:mm:ss)	Reduction and repair time (hh:mm:ss)	Total approach, ports placement and repair time (hh:mm:ss)
1	Scope used	3.8	00:08:22	8.9	00:04:55	00:08:34	00:15:33	00:37:24
2	Needle arthroscope, 0°	4.9	00:04:27	8.1	00:06:35	00:05:58	00:49:38	01:06:38
3	Needle arthroscope, 0°	6.2	00:05:31	4.7	00:07:29	00:09:02	00:51:13	01:13:15
4	Needle arthroscope, 0°	5.1	00:06:12	10.4	00:08:11	00:09:14	00:45:26	01:09:03
5	Needle arthroscope, 0°	3.3	00:03:37	5.9	00:04:38	00:06:39	00:45:08	01:00:02
6	Needle arthroscope, 0°	4.0	00:05:09	5.4	00:07:22	00:12:04	01:10:14	01:35:49
7	Needle arthroscope, 0°	4.1	00:02:33	5.2	00:04:12	00:07:09	00:23:01	00:36:55
8	2.4 mm, 30°	4.0	00:04:04	6.5	00:07:21	00:08:24	00:46:12	01:06:01
9	2.4 mm, 30°	5.2	00:04:15	6.0	00:05:47	00:03:01	00:46:43	00:59:45
10	2.4 mm, 30°	4.2	00:03:57	7.0	00:04:48	00:09:28	00:32:55	00:51:08
Mean		4.48	00:04:49	6.81	00:06:08	00:07:57	00:42:36	01:01:36
±SD		0.85	00:01:37	1.81	00:01:26	00:02:26	00:15:25	00:17:23

**TABLE 6:** Surgical measurements and duration of fluoroscopic procedures.

Cadaver	Caudal approach length (cm)	Caudal approach time (hh:mm:ss)	Craniolateral approach length (cm)	Craniolateral approach time (hh:mm:ss)	Reduction and repair time (hh:mm:ss)	Total approach and repair time (hh:mm:ss)	Fluoroscopic images required	Permanent fluoroscopy time (min:s)
1	4.0	00:04:37	8.5	00:08:28	00:18:14	00:31:19	6	00:00:26
2	5.5	00:03:21	7.5	00:04:17	00:23:24	00:31:02	9	00:00:45
3	4.8	00:04:50	8.9	00:05:13	00:23:37	00:33:40	7	00:00:47
4	5.9	00:03:48	10.7	00:05:08	00:32:51	00:41:47	7	00:01:08
5	4.3	00:02:42	6.0	00:03:45	00:22:55	00:29:22	5	00:00:43
6	3.8	00:02:39	5.1	00:03:17	00:21:24	00:27:20	5	00:00:12
7	3.9	00:02:55	5.0	00:04:13	00:55:24	01:03:32	14	00:01:29
8	3.3	00:04:13	4.8	00:05:56	00:38:40	00:48:49	7	00:00:31
9	4.0	00:04:59	6.1	00:06:24	00:48:42	01:00:05	9	00:00:48
10	4.0	00:03:21	7.5	00:03:55	00:24:54	00:31:10	8	00:00:15
Mean	4.35	00:03:45	7.01	00:05:04	00:31:01	00:39:49	7.70	00:00:42
±SD	0.81	00:00:53	1.95	00:01:33	00:12:39	00:13:16	2.63	00:00:23

### 3.4 Fluoroscopy

**FIGURE 7:**
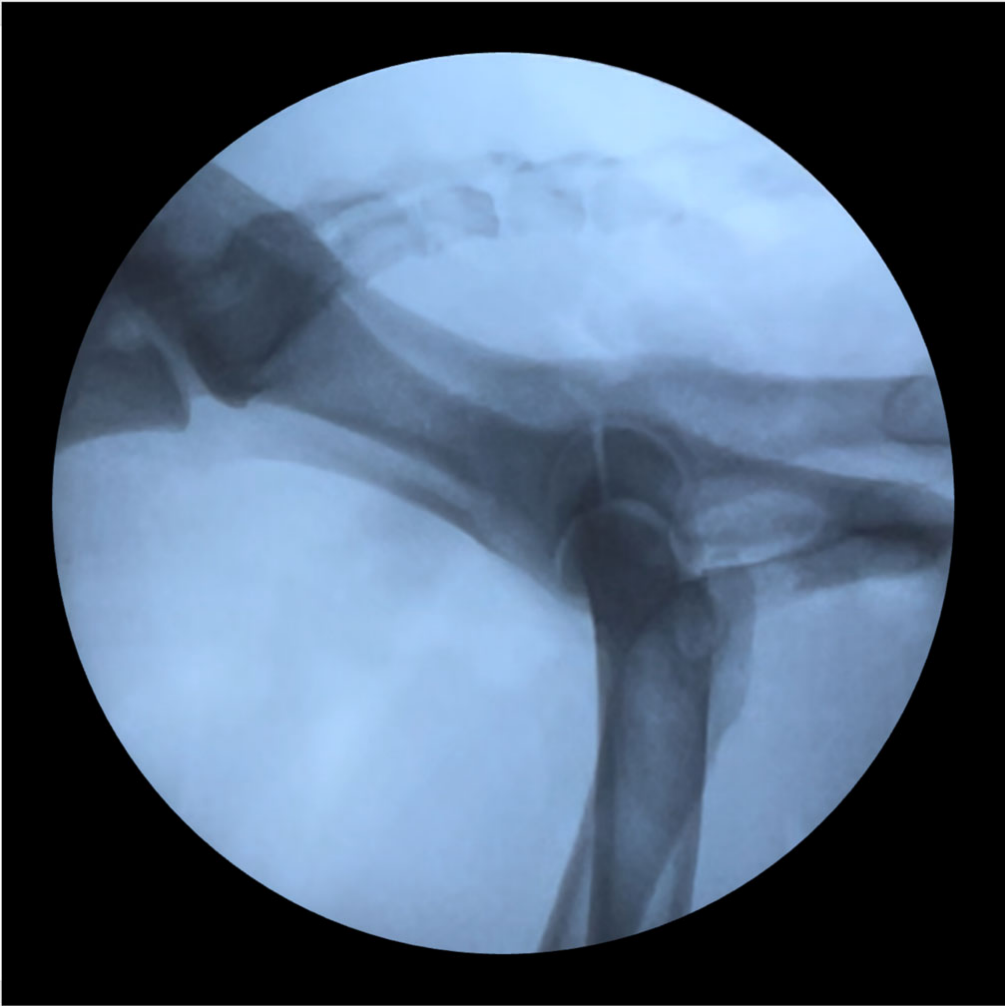
Fluoroscopic view of the acetabular fracture pre‐reduction.

On average, 7.7 fluoroscopic images were captured per procedure. Permanent fluoroscopy was used for an average of 42 s (±23) (Figure 7).

### 3.5 Arthroscopy

The median overall subjective visualization scores for arthroscopy were graded as good in three cases and excellent in seven cases. In two arthroscopy cases, visualization was obscured due to impingement of the scope by a fracture fragment during repositioning. Additionally, loss of the arthroscopic ports, requiring replacement, was documented in two cases.

### 3.6 Necropsy

**FIGURE 8:**
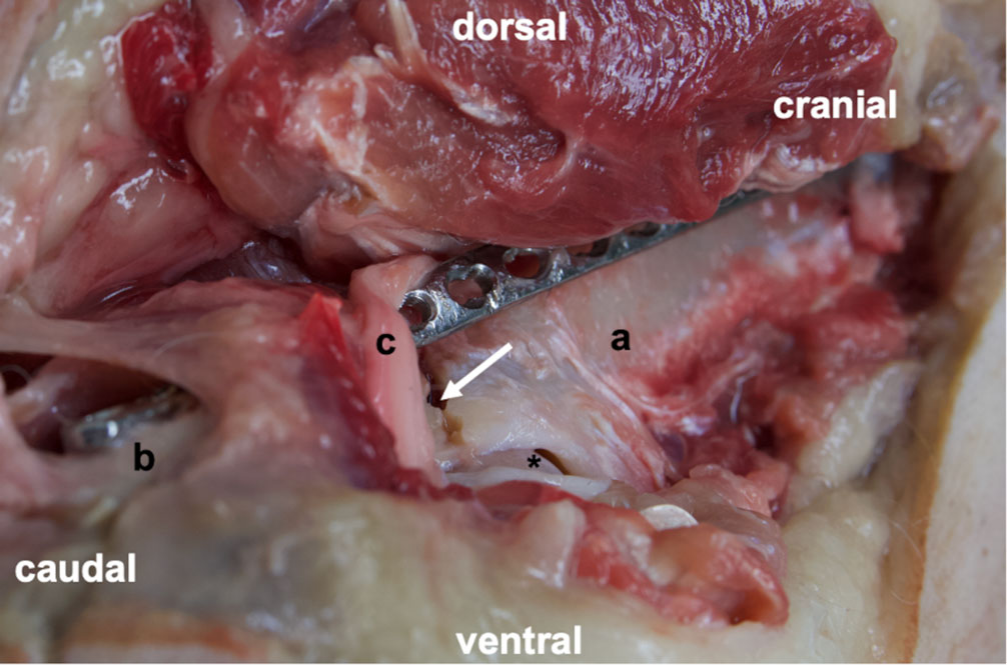
Necropsy post‐procedure of a right hip. The images are labeled as follows: “*” denotes the femoral head, “a” indicates the cranial part of the acetabulum, “b” marks the caudal part of the acetabulum, “c” marks the sciatic nerve over the locking compression plate and the arrow points at the fracture line.

In one case per group (2/20), a 2.7 mm LCP was placed above the sciatic nerve, which could have led to potential impingement of the nerve (Figure 8). No signs of nerve damage were observed on macroscopic inspection.

**FIGURE 9:**
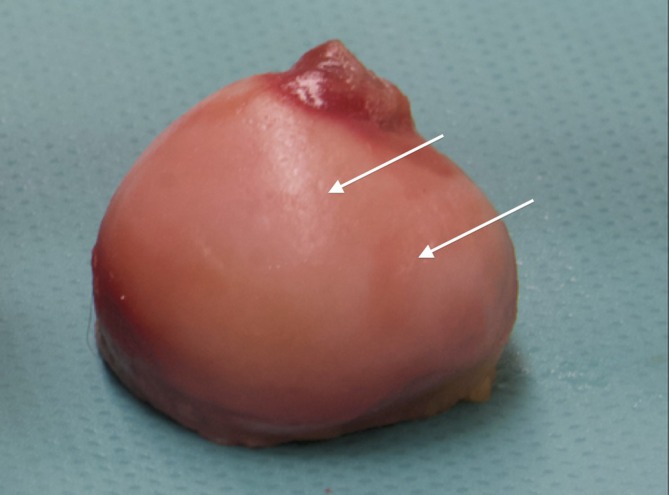
Cartilage damage of the femoral head. The arrows points at a superficial iatrogenic cartilage damage.

Iatrogenic cartilage damage was documented in six cases (6/10) and was categorized as superficial and minor (<10%) in all cases (Figure 9). Iatrogenic cartilage damage of the femoral head was seen in 6/10 cases, additionally cartilage damage to the acetabular surface was present in four cases (4/10). All damages were <10% in surface area. No difference was observed between standard and needle arthroscopy. All patients with intraoperative complications such as loss of the arthroscopic port or impingement of the scope between the femoral head and acetabulum during manual reduction of the fracture had iatrogenic cartilage damage.

## 4 DISCUSSION

An ideal fracture reduction method should be minimally invasive, accurate, rapid, and safe. Although the mean values for AA‐MIPO also meet the criteria for anatomic reconstruction, only 6/10 fractures were classified as anatomic and 4/10 as near‐anatomic. In contrast, FA‐MIPO reductions met only near‐anatomic criteria on average, with 4/10 classified as anatomic, 5/10 as near‐anatomic, and 1/10 as non‐anatomic. Therefore, we accept our first hypothesis.

We used a modified version of the classification system established by Boswell et al. to assess the quality of reduction, with fracture gap, step formation and medio‐lateral displacements <1 mm and small changes in pelvic angulation (<5°) being classified as anatomic reconstruction.^24^ However, studies investigating humeral condyle fractures have used a more stringent classification, defining anatomic reduction as 0 mm displacement, with malreduction graded as minimal (<1 mm), moderate (1–3 mm), and severe (>3 mm).^25^, ^26^ Whether the radiographic thresholds used in this study truly represent an adequate restoration of the articular surface remains debatable. While these thresholds are widely applied in orthopedic trauma surgery, clinical evidence supporting their validity for acetabular fractures is limited. Furthermore, the prognostic significance of specific gap and step measurements for long‐term clinical outcomes has not been definitively established. In human orthopedics, step defects are generally considered less tolerable than gaps, with CT‐based studies identifying critical thresholds of 1 mm for steps and 5 mm for gaps in predicting conversion to total hip arthroplasty (THA).^27^ Step defects >2 mm have also been associated with an increased risk of THA.^28^ Supporting this, Llinas et al. demonstrated in a rabbit model that step‐offs exceeding the local articular cartilage thickness (~1 mm) hinder effective cartilage repair, suggesting that anatomic reduction ideally requires step defects smaller than the cartilage thickness to optimize healing.^29^

Compared to conventional open reduction and internal fixation (ORIF), AA‐MIPO yielded fracture reduction quality comparable to published ORIF outcomes, whereas FA‐MIPO resulted in less precise reductions.^4^, ^7^, ^24^ Notably, reduction quality in this study was assessed by CT, in contrast to intraoperative visual assessment in Boswell et al.^24^ We believe that the principal advantage of MIPO may lie in enhanced visualization of the articular dome.^24^ Prospective clinical studies are needed to evaluate the effects of minimally invasive techniques on morbidity, postoperative pain, and healing time. When comparing our results to a previously published study evaluating FA‐MIPO in five canine cadavers and one clinical case, which reported consistently small fracture gaps (<2 mm), minimal step defects (<1 mm), and low coronal angulation (<5°), all AA‐MIPO cases in our study (10/10) met these criteria.^16^ In contrast, only seven of 10 FA‐MIPO cases in our study achieved comparable reduction quality. This discrepancy may be attributable to the absence of a mini‐arthrotomy in our FA‐MIPO protocol.

The time required for FA repair was similar to that reported previously.^16^ The time of surgical reduction, and repair from the first manipulation under fluoroscopic or arthroscopic assistance to the placement of the last screw comprised a median of 31 min in the FA group compared to 42 min in the AA group (*p* = .07). However, total procedural time, excluding the approach, and total surgical time were significantly longer in the AA group compared to the FA group. Therefor we accept the second part of our second hypothesis. Minor cartilage damage was common in AA‐MIPO, leading to acceptance of the first part of our second hypothesis.

Potential iatrogenic sciatic nerve injury was observed in 10% (2/29) of the procedures: one in the arthroscopic group and one in the fluoroscopic group. In both cases, the LCP was positioned superficial to the sciatic nerve rather than in direct apposition to the bone. Although no macroscopic damage was detected at necropsy, such malpositioning may predispose to nerve impingement in a clinical setting. It must be noted that the cadaveric model limits assessment of functional and microscopic nerve injury. This underlines the importance of meticulous periosteal tunneling to ensure plate‐to‐bone contact. Further investigations in live animal models are warranted to assess the clinical relevance of this finding. It is unlikely that the choice of procedure (AA‐MIPO vs. FA‐MIPO) influences the occurrence of impingement or damage to the sciatic nerve. This complication results from the minimally invasive surgical approach used during the creation of the periosteal tunnel and insertion of the LCP. Approximately 20% of the dogs (5/24) exhibited neurological deficits following open, direct surgical repair of acetabular fractures. It must be acknowledged that these deficits may have been caused by the initial trauma rather than the surgical intervention itself.^4^ We believe that a MIPO approach with reduced retraction of the femur, compared to an open reduction and internal fixation (ORIF) approach, might result in less sciatic nerve damage. Nevertheless, clinical studies are necessary to compare MIPO with traditional open approaches in terms of sciatic damage and neurological deficits. Damage to the major gluteal artery due to the misplacement of the arthroscopic portals was not observed in any arthroscopic procedure, which led to the assumption that arthroscopy is a safe technique.

One limitation inherent to this cadaveric study was the inability to observe extravasation into the surrounding tissues, which could lead to potential complications in living patients. Case reports in human medicine indicate that hip arthroscopic procedures for acute or healing acetabular fractures can result in intra‐abdominal compartment syndrome, a potentially life‐threatening complication.^30^ Coxofemoral arthroscopy has been documented as a minimally invasive technique for assisted toggle rod stabilization in hip luxations, as well as a diagnostic procedure prior to pelvic osteotomies and seems to be a safe procedure.^22^, ^31^, ^32^, ^33^, ^34^ The impact of an acetabular fracture and subsequent extravasation into the pelvic canal remains unclear and warrants further investigation.

Additionally, although AA‐MIPO allows for direct assessment of articular surface reduction, it is associated with several limitations. These include increased technical demands, a long learning curve, and prolonged operative times compared to fluoroscopy‐assisted techniques. The need for advanced arthroscopic skills and specialized equipment may further restrict its widespread implementation in clinical practice.

Another limitation of the study was the use of different scopes, specifically the 2.4 mm 30° arthroscope and the needle arthroscope. The decision was based on availability; however, this variability could potentially influence the degree of iatrogenic cartilage damage and quality visualization. Further studies are necessary to evaluate the effect of different scopes on these factors.

To provide a better overview during arthroscopy, manual distraction was performed by slight abduction and pulling of the affected hindlimb. In some cases, the distraction resulted in dislocation of the fracture, making reduction under arthroscopic visualization significantly more challenging. In our experience, it proved advantageous to position the arthroscope between the femoral head and the fragment connected to the ligament, hold it firmly in position, and bring about a reduction of the fracture by mobilizing the “loose” fragment. In some fractures, it was no longer possible to visualize the entire acetabular rim and acetabular notch once the fracture was reduced; therefore, control of the reduction had to be limited to visualization of the dorsal acetabular rim. In such cases, the combination of fluoroscopy and arthroscopy could be advantageous over arthroscopy alone.

The use of distractors for arthroscopy has been described for human coxofemoral arthroscopy, and in veterinary medicine, the use of distractors significantly enhances surgeon performance and decreases the incidence of iatrogenic cartilage damage in small animal hip arthroscopy, as opposed to relying on manual traction.^30^, ^35^, ^36^ Therefore, further studies are necessary to evaluate the feasibility and potential challenges when using distractors in AA‐MIPO of acetabular fractures, as well as differences in iatrogenic cartilage damage and visualization in comparison to arthroscopy performed without distractors.

A potential benefit of using arthroscopy instead of fluoroscopy to perform MIPO is the reduction in cumulative occupational radiation to the surgical staff. Although only limited data is published focusing on potential health implications for the surgical staff in human medicine, a reduction in radiation exposure (“as low as reasonably achievable”) should be sought whenever possible.^37^

Another limitation of MIPO techniques in general is associated to the configuration of fractures. Acetabular fractures with multiple fragments, comminuted fractures, or acetabular fractures with concurrent injuries such as iliac fractures may require an open approach to manipulate the fragments. Nevertheless, anatomic reconstructable, non‐comminuted acetabular fractures appear to occur most commonly in the central part of the acetabulum, which is why this fracture model was selected for the current study.^4^

In conclusion, arthroscopy‐assisted minimally invasive repair of simple acetabular fractures was feasible in all cases. No significant differences in terms of gap and step formation, as well as pelvic angulation were observed between arthroscopy and fluoroscopy; nevertheless, the mean medio‐lateral displacements were anatomic for arthroscopy‐assisted procedures and near‐anatomic for fluoroscopy‐assisted procedures. Further studies and clinical experience are necessary before recommending AA as an alternative technique to FA minimally invasive osteosynthesis or open approaches.

## AUTHOR CONTRIBUTIONS

Huels NH, TA, FTA, DrMedVet: Responsible for conception of the study, design of the 3D hemipelvis and preparation of the manuscript. Siedenburg J, TA, DrMedVet, DECVS: Responsible for design modifications, photographic documentation and statistical analysis. Huels NH, TA, FTA, DrMedVet and Siedenburg J, TA, DrMedVet, DECVS: Performed the preparation of the specimen, the precontouring of the locking compression plates and the surgical procedures. All authors reviewed, edited, and gave final approval to the manuscript.

## FUNDING INFORMATION

None to declare.

## CONFLICT OF INTEREST STATEMENT

The authors declare no conflicts of interest.
